# Exploration of Alloying Elements of High Specific Modulus Al–Li Alloy Based on Machine Learning

**DOI:** 10.3390/ma17010092

**Published:** 2023-12-23

**Authors:** Huiyu Li, Xiwu Li, Yanan Li, Guanjun Gao, Kai Wen, Zhihui Li, Yongan Zhang, Baiqing Xiong

**Affiliations:** 1 State Key Laboratory of Nonferrous Metals and Processes, China GRINM Group Co., Ltd., Beijing 100088, Chinagaoguanjun@grinm.com (G.G.); wenkai@grinm.com (K.W.); lzh@grinm.com (Z.L.); zhangyongan@grinm.com (Y.Z.); xiongbq@grinm.com (B.X.); 2 GRIMAT Engineering Institute Co., Ltd., Beijing 101407, China; 3 General Research Institute for Nonferrous Metals, Beijing 100088, China

**Keywords:** Al–Li alloys, machine learning, specific modulus prediction, composition optimization

## Abstract

In the aerospace sector, the development of lightweight aircraft heavily relies on the utilization of advanced aluminum–lithium alloys as primary structural materials. This study introduces an investigation aimed at optimizing the composition of an Al-2.32Li-1.44Cu-2.78Mg-0.3Ag-0.3Mn-0.1Zr alloy. The optimization process involves the selection of alloying elements through the application of machine learning techniques, with a focus on expected improvements in the specific modulus of these alloys. Expanding upon the optimization of the benchmark alloy’s components, a more generalized modulus prediction model for Al–Li alloys was formulated. This model was then employed to evaluate the anticipated specific modulus of alloys within a virtual search space, encompassing substitutional elements. The study proceeded to validate six Al–Li alloys with a notably high potential for achieving an improved specific modulus. The results revealed that an alloy incorporating 0.96 wt.% of Ga as a substitutional element exhibited the most favorable microstructure. This alloy demonstrated optimal tensile strength (523 MPa) and specific modulus (31.531 GPa/(g·cm^−3^)), closely resembling that of the benchmark alloy. This research offers valuable insights into the application of compositional optimization to enhance the mechanical properties of Al–Li alloys. It emphasizes the significance of selecting alloying elements based on considerations such as their solid solubility thresholds and the expected enhancement of the specific modulus in Al–Li alloys.

## 1. Introduction

The Al–Li alloy emerged as a remarkable engineering material, making its debut in the aerospace industry during the late 1960s [1]. Over the years, Al–Li alloys have garnered widespread acclaim, owning to their exceptional qualities, which encompass superior specific strength, exceptional fatigue resistance, high stiffness, and corrosion resilience. These attributes render Al–Li alloys exceptionally well-suited for aerospace applications, offering the advantage of lighter aircraft structures, leading to enhanced fuel efficiency and overall improved performance [2,3,4]. In the present day, the utilization of Al–Li alloys extends beyond the aerospace sector, finding applications across a diverse range of industries [5,6]. Given their myriad advantages, the future holds the promise of even greater adoption of Al–Li alloys across a multitude of applications [7].

Recently, another important metric has emerged as a critical factor for material selection in the aerospace industry. Specific modulus is a significant factor in applications that require high stiffness combined with low weight. In applications where weight is a critical factor, such as aircraft components, high specific modulus materials can better maintain structural integrity while minimizing weight. When improving the overall performance of aluminum alloys and achieving weight reduction in structural components, increasing the specific modulus of the alloy is an effective approach that is valuable in improving the overall performance of aerospace equipment and reducing fuel consumption [8,9]. In addition, the specific modulus is a critical consideration for many aerospace applications because it is directly proportional to the fundamental frequency at which a material will resonate. Vibrations can cause fatigue in aircraft structures, leading to catastrophic failures, so it is imperative to use materials with high specific modulus to reduce the likelihood of resonance [1,10,11,12]. Admittedly, the improved specific modulus of Al–Li alloys can enable the light-weighting of existing designs of the development of more efficient designs that would not be possible with traditional aluminum alloys.

Our previous studies have demonstrated the potential of achieving the design of high specific modulus aluminum–lithium alloys through the manipulation of the alloy [13]. The chemical modification of aluminum alloys has garnered significant attention [14], with numerous chemical elements such as Sr, La, and Er being widely researched for their applicability in alloying aluminum alloys [15,16,17,18,19,20,21]. In order to investigate the synergistic effect of Sr and La on the microstructure and mechanical properties of A356.2 alloy, Qiu [19] prepared an Al-6Sr-7La master alloy and studied its optimal content as well as microstructure and phase composition. As a result of microstructure refinement, the tensile strength, yield strength, elongation, and hardness of the alloy were improved by 13.8%, 7.7%, 52.4%, and 6.4%, respectively, compared to the conventional alloy. Wen [22] investigated the effects of Er on the microstructure and mechanical properties of the Al–Mg–Mn–Zr alloy. The addition of 0.4 wt.% Er led to the formation of primary Al_3_Er and refinement of the grain size of the cast alloy. The homogenized primary Al_3_Er decomposed into dispersed secondary Al_3_Er particles, resulting in an enhancement in the alloy’s strength, particularly at high temperatures.

Machine learning is a rapidly growing field that integrates computer science, statistics, and artificial intelligence, and has recently found extensive applications in materials design [23]. The technology enables the creation of intelligent algorithms that can learn and make predictions by analyzing large datasets, allowing researchers to predict material behavior, optimize processing techniques, and even design new materials with tailored properties. With increasing demands for advanced materials in various industries and applications, machine learning has become an essential tool for researchers to accelerate the discovery and development of novel materials [24]. Machine learning has various applications in the field of materials design, including property prediction, material discovery, process optimization, and the design materials with specific functionalities [25]. By building databases, predicting material properties, optimizing preparation, and processing parameters, machine learning can accelerate the discovery of new materials and improve material performance, such as thermal conductivity [26], electrical conductivity [27], mechanical strength [28], and hardness [29]. Specifically, the utilization of machine learning holds immense potential for designing materials with tailored properties. Wen [30] leveraged a learning model in conjunction with experimental design algorithms to identify high-entropy alloys with enhanced hardness in the Al–Co–Cr–Cu–Fe–Ni multi-component system. Remarkably, they produced several alloys exhibiting hardness values exceeding the best value in the initial training dataset by 10% after conducting just seven experiments, thus achieving the desired performance. In this context, machine learning is rapidly transforming the field of materials design, and its continued development holds significant promise for the creation of more advanced and cutting-edge materials.

In our previous study, a new type of high-performance Al–Li alloy (Al-2.5Li-1.4Cu-2.6Mg-0.3Ag-0.3Mn-0.1Zr) with high specific strength and specific modulus was obtained. In this study, we explored the potential of substitutional elements and additional elements to improve the composition of an alloy by a machine learning method. The solid solubility of each element in aluminum alloys was determined, and a virtual alloy search space was established to screen for new high specific modulus Al–Li alloys. Potential additives in Al–Li alloys were investigated, and the microstructure characteristics of high Al–Li alloys were studied. The influence mechanism of a substitutional or additional element on the specific modulus of alloys was analyzed through the microstructure of the alloy. This work explores possible new alloying elements in high performance Al–Li alloys in combination with a materials database and machine learning methods. Correspondingly, the experimental verification results are interpreted from a guiding perspective of model prediction.

## 2. Materials and Methods

### 2.1. Dataset Construction

The dataset was constructed by collecting data from various sources including experiments, standard datasets, and the literature. Experimental data were collected by synthesizing and characterizing materials, which involved measuring various physical, chemical, and mechanical properties. Standard dataset and literature mining involved searching through scientific publications to extract relevant data on materials’ properties.

After conducting the data collection process, a total of 145 data points were initially obtained. Furthermore, experimental validation in our previous study [13] led to the successful acquisition of six additional data specifically about Al–Li alloys, which were subsequently integrated into the dataset. As a result, the final dataset comprised 151 alloy samples, rendering it a more extensive and comprehensive representation of the target material compositions. Table 1 presents the categories of elements included in the dataset, as well as the range and quantity of each element’s concentration.

### 2.2. Feature Space Construction

In light of the absence of a definitive physical and chemical basis to account for the variability of elastic modulus in Al–Li alloys, a comprehensive description of potential influencing factors is necessary to achieve more accurate predictions. Informed by the results of literature reviews and data analyses, this study posits that the elastic modulus of Al–Li alloys can be predicted by incorporating elemental characteristic parameters and elemental atomic fractions. Elements possess various characteristic parameters, including atomic basic properties, chemical properties, and physical properties. Table 2 presents the definitions, symbols, units, and data types of all elemental parameters in the dataset, as well as the abbreviations of the parameters used in the subsequent construction of the characteristic space.

The elemental compositions and properties are characterized by a multitude of parameters, including atomistic fundamental properties, chemical aspects, and physical traits. To explore the potential relationship between these parameters, the dataset’s weight percent (wt.%) is first converted to atomic percent (at.%), and then the characteristic candidate features are calculated using Equations (1)–(4). Based on these features, a descriptive feature space related to the specific modulus is constructed, serving as the initial characterization of the alloy.

(1) $$x_{1}=\sum_{i=1}^{N}a_{i}x_{i}$$

(2) $$x_{2}={\left(\sum_{i=1}^{N}\frac{a_{i}}{x_{i}}\right)}^{-1}$$

(3) $$xd=\sqrt{\sum_{i=1}^{N}a_{i}{\left(1-\frac{x_{i}}{x_{1}}\right)}^{2}}$$

(4) $$xr=\text{max}[a_{i}{\left(1-\frac{x_{i}}{x_{1}}\right)}^{2}]-min[a_{i}{\left(1-\frac{x_{i}}{x_{1}}\right)}^{2}]$$

where $a_{i}$ is the atomic fraction and $x_{i}$ is the elemental characteristic parameter.

To ensure that each feature has an equal contribution to this analysis and to avoid the dominance of certain features in the modeling process, the standardization method was applied to each feature candidate by subtracting the mean and dividing by the standard deviation, as shown in Equation (5).

(5) $$x^{*}=\frac{x-\mu }{\sigma }$$

where μ is the mean value and σ is the standard deviation.

### 2.3. Machine Learning

Feature filtering is a common approach used in machine learning to enhance the performance of a model by selecting the most informative features out of the available set of features. This study implements a three-step process to filter candidate features, including Pearson correlation coefficient filtering (PCC) [31], recursive feature elimination (RFE) [32], and best subset selection (BSS) [33].

The PCC, also referred to simply as the correlation coefficient, constitutes a statistical measure that evaluates the strength and direction of a linear association between two continuous variables. It assumes values ranging from −1 to 1, where a coefficient of −1 signifies a perfect negative correlation, 0 indicates no correlation, and 1 suggests a perfect positive correlation.

RFE is a feature selection algorithm that is widely used in the field of machine learning and data analysis. RFE utilizes a recursive approach to iteratively eliminate the least significant features until the desired number of features is reached. It employs a machine learning algorithm to assess the significance of each feature in the feature set, which helps to identify the most important features for the model.

BSS involves exploring all possible feature subsets to identify the optimal set that will lead to the best performance of the model. This method plays a significant role in feature filtering as it helps to reduce the number of features and prevent overfitting, thereby improving the generalization of the model.

AdaBoostRegressor is a popular ensemble learning algorithm, which combines the predictions of multiple weak regressors to produce a robust and accurate model. The algorithm iteratively trains a series of weak models, each of which is focused on the difficult data points that were classified by the preceding model. By combining the results of these individual models, AdaBoostRegressor achieved a more accurate and stable prediction, reducing the influence of outliers and noise within the data.

Mean squared error (*MSE*) calculates the average of the squared differences between predicted and actual values and is typically used to evaluate the performance of regression models, and is given by Equation (6),

(6) $$MSE=\frac{1}{n}\sum_{i=1}^{n}{\left(y_{i}-{\hat{y}}_{i}\right)}^{2}$$

where $y_{i}$ is the actual value of the sample and ${\hat{y}}_{i}$ is the predictive value. A lower *MSE* indicates a better performance of the model in predicting the target variable. The determination coefficient (*R*^2^) is a statistical measure commonly employed in regression analysis to quantify the ability of the regression model to explain the data and the predictive power of the independent variables concerning the dependent variable, and is given by Equation (7),

(7) $$R^{2}=\frac{\sum_{i=1}^{n}{\left({\hat{y}}_{i}-{\bar{y}}_{\;}\right)}^{2}}{\sum_{i=1}^{n}{\left(y_{i}-{\bar{y}}_{\;}\right)}^{2}}$$

*R*^2^ values fall between 0 and 1, where values closer to 1 indicate a better fit of the regression model to the data, while values closer to 0 suggest a poorer fit.

### 2.4. Experiments

The experimental alloy ingots were obtained via the process of melting and casting, with a diameter of 133.5 mm and a height of approximately 200 mm for each ingot. The elastic modulus of the samples was determined using the dynamic testing method with an RFDA-HT 1050 instrument (IMCE, Leuven, Belgium). The samples were meticulously fabricated into rectangular blocks with dimensions of 75 mm × 25 mm × 5 mm, ensuring homogeneity and plane-parallelism of the material, with a transverse axis dimensional variation of no more than 0.7% and a surface devoid of any defect. To derive the specific modulus of the samples, it is imperative to conduct a density measurement of the samples using Archimedes’ principle, also known as the buoyancy method. The tensile test was conducted on a CMT4304 microcomputer-controlled electronic universal testing machine (MST, Eden Prairie, MN, USA) at testing of 2 mm/min. The tensile strength was determined from the stress–strain (σ–ε) curve. The scanning electron microscope used in this study was JEOL JSM 7001F (JEOL, Tokyo, Japan), which was equipped with an Energy Dispersive Spectrometer (EDS). Scanning tests allowed for observation of the morphology of the second phase present in the sample, with EDS-enabled analysis of the component composition of phases. The morphology and texture of the precipitated phase in the alloy were characterized using Tecnai G2 F20 S-TWIN Transmission Electron Microscopy (TEM) (FEI, Hillsboro, OR, USA). The samples were dual-beam thinned using a magnetically driven system, with a composition ratio of V(Nitric acid):V(Methanol) = 1:3 for the dual-beam liquid.

## 3. Results and Discussion

### 3.1. Elements Filtering

To explore the feasibility of melt-processing and casting aluminum alloys, the solubility of elements considered for aluminum alloy compositions was investigated. Literature research was conducted to compare the solubility of component elements with the calculations obtained from the Al–M binary phase diagram. To expand the search space for Al–Li alloys with higher specific modulus, the maximum solubility of each element was determined by selecting the larger solubility value between the literature and the calculation data and is expressed in weight percent (wt.%), as reported in reference [34].

To further expand the search space, it is essential to evaluate the possibility of other elements with solid solubility in aluminum as potential alloying elements in Al–Li alloys. Among the lanthanide elements present in the dataset, Ce emerged as a promising candidate [35]. Additionally, researchers suggested that Ca [36], Sr, La [19], Pr, Nd [37], Sm [18], Ho [38], Er [22], Gd [39], Eu [17], and Yb [20] have been utilized in aluminum alloy micro-alloying. Interestingly, La, Pr, Nd, Sm, Ho, Er, Gd, Eu, and Yb are lanthanide elements, indicating their potential as alloying elements in aluminum alloys. The maximum addition mass percentage was determined by extracting the actual maximum alloy composition value reported. These steps have allowed for the identification of promising additive elements that could enhance the performance of Al–Li alloys. The number of candidate alloying elements has been increased to 34, and Table 3 presents the solid solubility of each of these elements in the Al matrix, with units in wt.%. The elements with a solid solubility greater than or equal to 0.5 wt.% are classified as substitutional elements (M1), replacing Li, Cu, and Mg in the benchmark alloy. The elements with a solid solubility lower than 0.5 wt.% are referred to as an additive element (M2), which is directly incorporated into the benchmark alloy as the trace element. In conclusion, a total of 17 M1 were determined as viable options, which included Zn, Ag, Ga, Ge, Ho, Si, Sm, Mn, Ti, Ce, La, Cr, Sn, Er, Sr, V, and Ca. Furthermore, 17 M2 candidates were also identified, such as Au, Gd, Yb, Sb, Ni, Nb, In, Nd, Sc, Bi, Zr, Be, Eu, Pr, Y, W, and Fe. These findings showcase a promising range of alternatives to the traditional elements used in the field.

### 3.2. Model Construction

**Figure 1:**
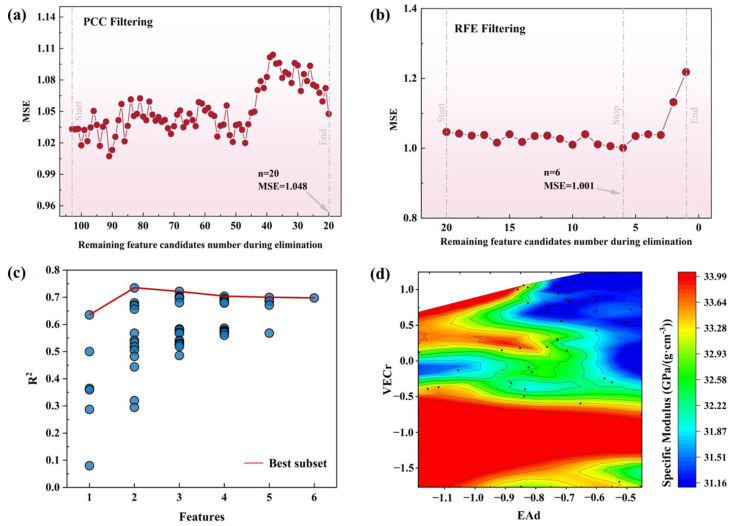
Results of feature filtering: (**a**) Feature filtering by Pearson correlation coefficient of the initial feature candidates. (**b**) Feature filtering by recursive elimination. (**c**) Feature filtering by best subset selection. (**d**) Features EAd and VECr in relation to specific modulus.

The first step was to use the Pearson correlation coefficient for feature filtering with a threshold of 0.9 [40]. AdaBoostRegressor [41] from the sklearn ensemble model was selected, and 10-fold cross-validation [42] with *MSE* was used to evaluate the model accuracy. The *MSE* of the model after each step of feature selection was statistically analyzed and is shown in Figure 1a. After the initial filtering process, a set of 20 features was obtained: NA1, TM1, G1, C1, R1, VL1, EA1, C2, EA2, HFd, EAd, HFr, ICr, EAIr, Cr, Rr, VLr, ENr, EAr, and VECr. The correlation coefficients between all these features were found to be below 0.9.

During the feature filtering process of the second step, the sklearn ensemble model GradientBoostingRegressor was selected using an ML model that evaluated the scores of each model and the base learning algorithm of RFE. The model evaluation approach involved a 10-time and 10-fold cross-validation method, with an *MSE* evaluation score. The final feature number for comparison was set to 1–20, based on 20 cycles of model evaluation values. The results, as presented in Figure 1b, revealed that the *MSE* evaluation score was the lowest when the remaining feature number was 6. Thus, 14 features were eliminated, and 6 features were retained, namely, HFd, EAd, ICr, Cr, Rr, and VECr in the second step of the feature selection process.

In the third step of the feature selection process, we utilized the BSS technique to narrow down the set of feature candidates. The AdaBoostRegressor algorithm was selected as the machine learning algorithm, with default parameter values (estimators = 50, learning_rate = 1). To evaluate the overall performance of the models generated by AdaBoostRegressor, we employed a 10-time and 10-fold cross-validation method, with *MSE* and *R*^2^ as the evaluation metrics. The evaluation results of the 26 models are depicted in Figure 1c. The results indicate that the best fitting performance was obtained when there were only two remaining features, namely, EAd and VECr.

EAd represents the extent of deviation in the electron affinity of constituent elements in alloys, whereas VECr represents the degree of mismatch in the valence electron count of these chemical elements. The relationship between these two prominent features and the specific modulus of Al–Li alloys is illustrated in Figure 1d. According to the available raw data, it can be inferred that the specific modulus of Al–Li alloy is significantly affected by the electron affinity and valence electron count among its constituent elements. Specifically, a larger specific modulus is typically observed when the deviation in electron affinity is reduced and the discrepancy in valence electron count is comparatively high. These findings imply that a thorough understanding of the electronic structure and chemistry of constituent elements is imperative for the optimization of the mechanical properties of alloys.

**Figure 2:**
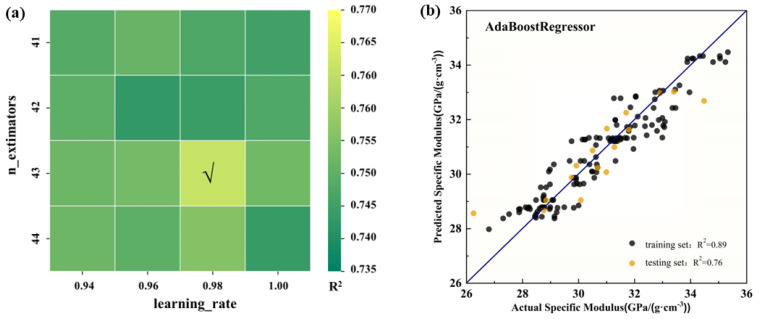
(**a**) Optimization of prediction model parameters. (**b**) Comparison of the predicted values and measured values based on the optimized model.

To optimize the accuracy of the ML model, parameter tuning was conducted on the AdaBoostRegressor. The *R*^2^ value of the testing set was utilized as the metric for parameter selection, with higher *R*^2^ values being preferred, while maintaining a testing set ratio of 0.1 to ensure the reliability of the model. The tuning parameters for the AB model were the learning_rate and n_estimators. Through parameter tuning, we were able to significantly improve the model’s evaluation performance, as demonstrated by the results presented in Figure 2a. The optimal values that we determined for the learning_rate and n_extimators were 0.98 and 43, respectively, and the optimized set of parameters was utilized to predict the specific modulus of Al–Li alloys. The evaluation results showed a strong correlation between the original specific modulus measurements and the predictions generated by the optimized AB model, as evidenced by the *R^2^* values of 0.89 and 0.76 for the training and test sets, respectively, indicating a high level of predictive accuracy. The results of the model evaluation of the training set and testing set are presented graphically in Figure 2b.

### 3.3. Virtual Alloy Search Space Prediction

**Figure 3:**
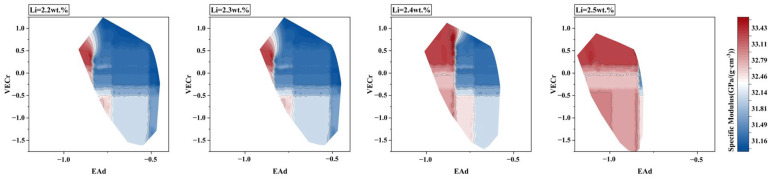
Specific modulus distribution of virtual alloys with different Li content.

By selecting the alloy Al-2.5Li-1.4Cu-2.6Mg-0.3Ag-0.3Mn-0.1Zr as the benchmark alloy, the present study aimed to investigate the effects of element M1, with a solid solubility greater than or equal to 0.5 wt.%, and direct addition of alloying element M2, with a solid solubility less than 0.5 wt.%, into the alloy. The elemental step setting values for adding M1 and M2 of the virtual alloy space were kept at 0.1 wt.%, while the upper value of the virtual composition of each element was set as the upper solubility limit. Furthermore, the solid solubility of the M1 element was rounded up to the nearest tenth decimal place. To mitigate the computational cost resulting from the addition of M2 elements to the virtual alloy, a multi-round predictive calculation was carried out to explore the correlation between the modulus and the mass fraction of the Li element. As shown in Figure 3, the results indicate that the possibility of a substantial increase in the specific modulus of Al–Li alloys occurs only when the mass fraction of Li exceeds 2.2 wt.%, which means that the substitution of the Li element was limited to below 0.3 wt.%. Consequently, restricting the content of the Li element was employed to constrain the prediction space and reduce the computational cost.

Based on the established specific modulus prediction model, the specific modulus of the reference alloy was predicted to be 33.137 m, which is used to compare the specific modulus of other alloys with the equivalent prediction condition. When only the substitutional element M1 exists in the alloy with no addition of the addictive element M2, the maximum predicted specific modulus of the alloy is 33.986 m. The maximum predicted specific modulus corresponds to M1 with different substitution elements, each of which contains a different substitution level. When the predicted specific modulus exceeds 33.137 m, the M1 elements that correspond to such a high specific modulus include Zn, Ag, Ga, Ge, and Ho, with substitution levels ranging from 2.8 to 3.9 wt.%, indicating that the observed high specific modulus of the alloy occurs only when the substitution level of the M1 element is relatively high in the search space.

When the M1 elements were Zn, Ag, Ge, and Ho, the Li content was found to be in the range of 2.3 to 2.5 wt.%, with the majority of the substitutional elements being distributed amongst Cu and Mg. The Li substitution level, on the other hand, varied between 0 and 0.2 wt.%. Interestingly, when Ga was substituted as the M1 element, the highest level of the Li content, 2.2 wt.%, was observed, indicating that Ga was a potential substitutional for Li in the Al–Li alloy. To investigate the extent to which Ga can replace Li in the alloy, a new visual dataset was established by replacing Li with Ga and varying the Li content in the range of 0–2.5 wt.%. A total of 10,799 data points were generated and sorted in descending order based on the predicted specific modulus. It was found that even at the highest specific modulus, the Li content remained at 2.2 wt.%, indicating a substitution level of only 0.3 wt.%. The results underscore the suitability of Ga as a replacement element for Li in the Al–Li alloy, albeit to a limited extent.

When element M2 was added as Be, the maximum predicted specific modulus of the virtual alloy attained its highest value of 33.137 m, which coincided with the predicted specific modulus of the benchmark alloy. Conversely, the incorporation of other trace elements did not yield a substantial improvement in the virtual alloy’s maximum predicted specific modulus, which remained at 32.980 m, lower than that of the benchmark alloy. It is suggested that trace element incorporation would not yield a significant improvement in the specific modulus of the Al–Li alloy.

When both M1 and M2 were present in the virtual alloy composition, the predicted specific modulus of the alloy data in the virtual alloy space was the same as that of the virtual alloy space containing only the substitutional element M1. The maximum predicted specific modulus for all substitutional and additional element configurations is 33.980 m, further demonstrating that low-mass fraction trace added elements have negligible impact on the specific modulus of Al–Li alloys.

### 3.4. Experimental Verification

To further identify novel alloys that satisfy the high specific modulus criterion in the prediction results, we integrated domain knowledge and expert experience for alloy composition screening. Based on this integration, four screening rules were established as follows: (1) The data include compositions containing M1 element Ag, which is already present in the benchmark alloy. (2) The data include compositions with the highest predicted specific modulus in the virtual alloy space. (3) The data include compositions with M1 content higher than Mg content, between Mg and Cu contents, and lower than Cu content in the benchmark alloy. (4) The data include compositions containing M1 elements such as Ag, Ho, Zn, Ga, and Ge. Based on these criteria, six Al–Li alloys with a high potential for specific modulus were identified from the search space and experimentally validated. The compositions and predicted specific modulus are presented in Table 4.

At the bottom of the ingots, samples of the as-cast alloy were obtained and subjected to elastic modulus and density measurements to determine the specific modulus. The cast performance of the alloy is presented in Table 5. Results from the tests indicated that the alloy with Ge as the supplementary element for M1 had the lowest elastic modulus and specific modulus, while the elastic modulus of the designed alloys with M1 replaced by Ho, Zn, Ga, and Ag exhibited a decreasing trend in that order.

**Figure 4:**
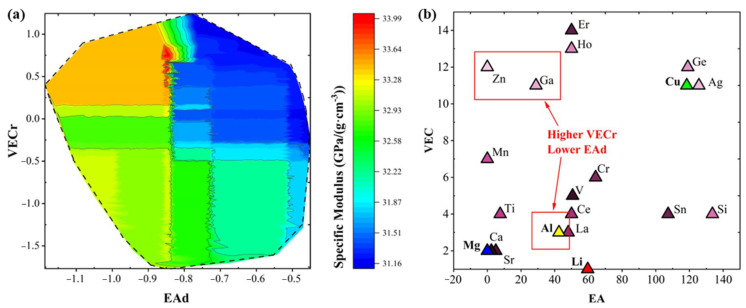
Filter the relationship between features and specific modulus and elements: (**a**) Relationship between screening features and specific modulus of Al–Li alloys. (**b**) Chemical parameters of substitutional elements.

We employed the machine learning Al–Li alloy-specific modulus prediction model to integrate filtered features. The virtual alloy modulus distribution presented in Figure 4a shows that the predicted specific modulus distribution of the virtual alloy is consistent with the original dataset except for the missing data points due to the uneven distribution of the dataset. This indicates that the established specific modulus prediction model can predict the missing data parts in the dataset with good performance, but the upper limit of the model performance is limited by the original dataset. Specifically, a larger VECr and a smaller EAd value may contribute to a higher modulus of the Al–Li alloy, and this observation confirms the high accuracy and reliability of the modulus prediction machine learning model and sheds light on the mapping relationship between the two datasets. Figure 4b depicts the correlation between the VEC and EA values of key constituents that act as substitutional element M1 in the Al–Li–Cu–Mg alloy. Our theoretical model has characterized that the addition of Zn, Ga, and Ho, exhibiting an elevated VECr and a reduced EAd, is instrumental in achieving a superior specific modulus of the alloy, aligning with the discernible variables highlighted in this research. The degree of mismatch in the chemical valence electron numbers of all elements of the alloy composition will increase if the elements Zn, Ga, and Ho participate in the alloying as the principal alloying elements, as shown in Figure 4b, which shows that the valence electron numbers of the alloying elements corresponding to the high specific modulus differ from those of the Al element. Correspondingly, the extent of deviation of the electron affinity of all the alloying components will decrease, and the electron affinity of the elements represented in Figure 4b are similar to those of the Al element.

Heat treatment of samples from the ingot bottom was carried out by DSC experiment to investigate the heat treatment regime for improving the mechanical properties of four alloys. Based on the experimental results, preliminary uniformization heat treatment regimens were identified and explored for the examined design alloys, as summarized in Table 6. To mitigate the potential influence of casting ingot quality on the elastic modulus and ensure that the design alloys are in the same metallurgical state as the alloys in the original dataset, the experimental alloys were subjected to extrusion deformation, solution quenching, and artificial aging heat treatment.

**Figure 5:**
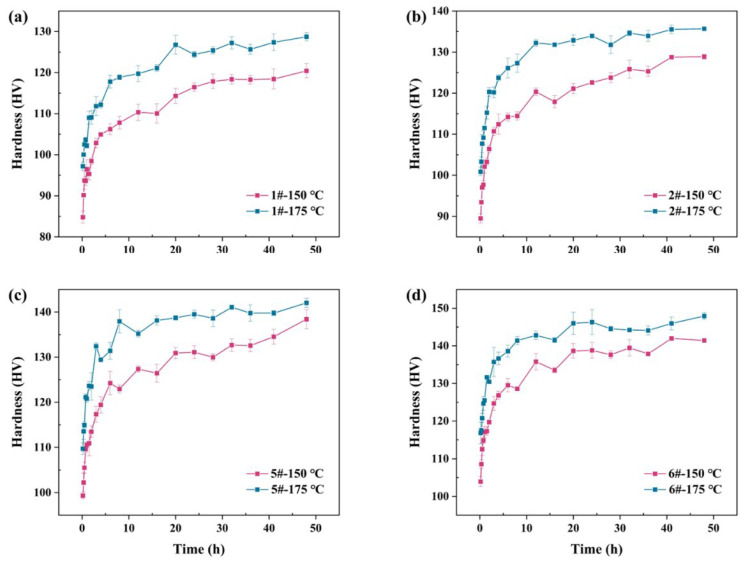
Aging hardening curves: (**a**) 1#. (**b**) 2#. (**c**) 5#. (**d**) 6#.

The designed alloy ingots subjected to uniform heat treatment were processed into extruded samples using the hot extrusion process, with the holding system set at 440 °C/4 h during extrusion. The extruded samples were subsequently subjected to solubilization heat treatment under the same regime to maintain their consistency with the reference alloy state. For the extruded samples, the solubilization heat treatment regime remained at 515 ℃/3 h, with subsequent quenching. As the compositionally optimized alloys contained multiple newly added elements, the optimal aging regime was explored. The aging heat treatment temperature was set at 150 °C and 175 °C and different aging treatments were applied to alloys 1#, 2#, 5#, and 6#. Figure 5 shows the aging hardening curves of the four alloys. The results show that the aging response rate at 175 °C is faster and the peak hardness is higher compared to 150 °C for all four alloys. The aging hardening curves show that the peak hardness of 1# alloy is achieved after a 20 h aging treatment, 2# alloy after a 12 h aging treatment, 5# after a 16 h aging treatment, and 6# after a 20 h aging treatment. To facilitate the cross-component comparison of alloy performance, a standardized aging regime of 175 °C/20 h was established and applied to all samples.

**Figure 6:**
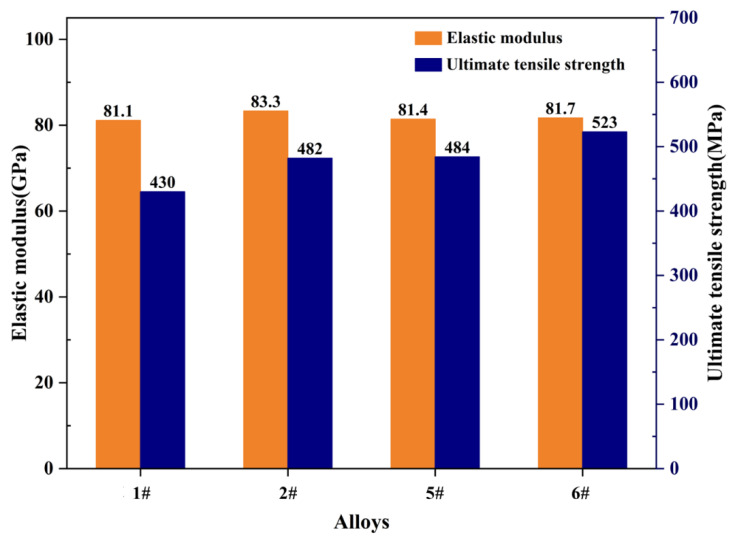
Elastic modulus and tensile strength of composition-optimized alloys.

To compare the performance of the compositional optimized alloys to the benchmark alloy, further investigations were carried out to test the strength of the compositional optimized alloys, disregarding density factors, and the results are presented in Figure 6. It is evident that the maximum elastic modulus of the 2# alloy, which employs Ho as a substitutional element, exceeds that of the benchmark alloy, whereas its strength is less than that of the benchmark alloy. Among the four compositional optimized alloys, the 6# alloy, which features Ga as a substitutional element, displays the highest strength, reaching 523 MPa, which is comparable to the ultimate tensile strength of the reference alloy (532 MPa). By combining density measurements with the evaluation of elastic modulus, the results of the specific modulus in the aged state are presented in Table 7. It can be observed that there is a moderate increase in the specific modulus of the aged state compared to the as-cast state for all four newly designed alloy compositions. The alloy with Ho as the substitutional element in the 2# alloy exhibited the highest specific modulus, achieving a value of 31.620 GPa/(g·cm^−3^), which is comparable to the specific modulus of the reference alloy (31.691 GPa/(g·cm^−3^)), thus hinting at a highly favorable response to the addition of new elements to the alloy design. Additionally, the specific modulus attained by the alloys containing Zn and Ga as the substitutional elements in the 5# and 6# compositions, respectively, are also noteworthy as they revealed a marginal difference of 0.1 m or less compared to 2# alloy. Compared to the testing results of the as-cast alloy, the prediction errors for the specific modulus of the aged alloy were reduced, with the maximum prediction error being −9.15% and the minimum prediction error being only −4.85%. It is worth noting that the specific modulus of designed alloys was determined based on measured density, which led to the model’s predicted specific modulus values being larger than the actual measured values, resulting in negative prediction errors for all cases.

**Figure 7:**
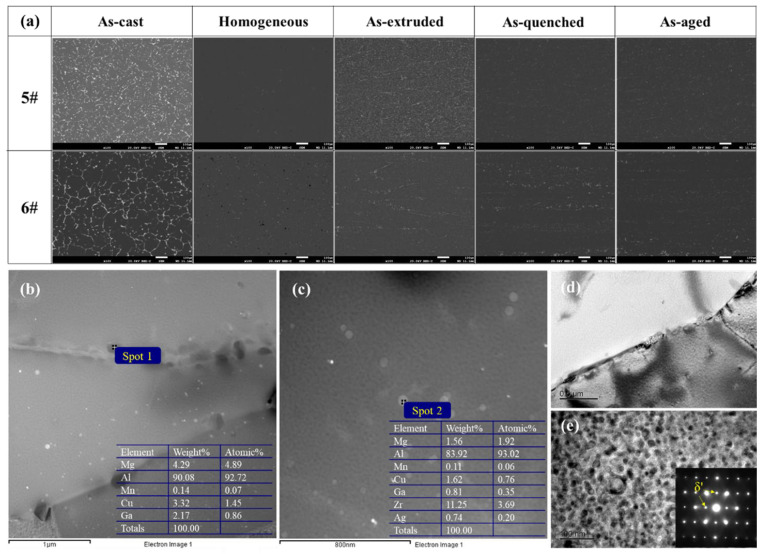
(**a**) Evolution of the second phase in design alloys from the as-cast to the aged state. (**b**,**c**) Spectrum processed images. (**d**,**e**) Bright-field image and diffraction spots.

SEM testing was employed to observe the microstructure of alloy samples at different states, namely, 5# and 6# alloys, exhibiting superior strength testing results following composition optimization. The evolution of the phase was analyzed, and the results are presented in Figure 7a. The scanning results at low magnification revealed that both 5# and 6# alloys exhibited network segregation in the as-cast state, which was essentially eliminated after homogenization heat treatments. Alloys 5# and 6# exhibited a uniform distribution of second phases following hot extrusion. After undergoing solid solution and quenching treatment, the second phase was not completely dissolved and no new large phase separation occurred during subsequent aging heat treatment. The composition of the second phase in the optimized alloy was analyzed using energy-dispersive X-ray spectroscopy (EDS) in different states. In alloy 5#, the main second phases consisted of two different components: phases containing Mn and phases containing Zn, with small size and dispersed distribution in the matrix. In alloy 6#, the main second phases comprised the AlCuMgMn quaternary phase and the AlCuMgGaAg quinary phase.

Among alloys 5# and 6# with optimized compositions, alloy 6# exhibited the least substitution of the surrogate element M, with a nominal replacement of only 1.1 wt.% of the Cu and Mg elements in the alloy being attributed to Ga. Figure 7b,c show the selected area electron diffraction pattern, TEM bright field image, and dark field image of alloy 6# after being subjected to aging treatment at 175 °C/20 h. According to the spectral processing image of the alloy, it can be observed that the strengthening phase containing Ga element in the alloy is mainly distributed near the grain boundaries (Spot 1), coexisting with Cu and Mg elements, while the size of the second phase is approximately 0.2 μm. Moreover, the strengthening phase of alloy 6# encompasses the Al_3_Zr phase with a diameter of approximately 50 nm, diffusely dispersed in the matrix (Spot 2). From Figure 7e, it can be observed that the predominant precipitate phase in the matrix is the small-sized, spherical δ’ precipitate phase [43], while there also exists a large-sized, shell-core structured spherical phase, the β’/δ’ composite phase [44], both of which contribute to the strength of the alloy.

## 4. Conclusions

Computer-assisted screening and experimental validation of alloying elements that have the potential to improve the specific modulus of Al–Li alloys were conducted with the help of machine learning in this work; they obtained an optimized design scheme for Al–Li alloys with better overall performance. The screening results of feature space engineering show that the designed Al–Li alloy may achieve the goal of high specific modulus when the degree of mismatch between the valence electron number of the principal alloying element and the base element Al increases, as well as the degree of deviation from the electron affinity of the Al element decreases.

In addition, the prediction results of the machine learning model on the virtual alloy space illustrate that the microalloying of trace elements has a negligible effect on the specific modulus of the Al–Li alloy. According to experimental validation results, the Al-2.68Li-3.40Ho-0.11Cu-0.37Mg-0.30Ag-0.32Mn-0.13Zr alloy with the addition of the element Ho was found to attain high elastic modulus of 83.3 GPa and high specific modulus of 31.620 m. Through the characterization of the microstructure evolution, it was determined that the Al-2.61Li-1.26Cu-1.81Mg-0.96Ga-0.29Ag-0.31Mn-0.14Zr alloy with the addition of the element Ga has the best organization and the highest specific strength and specific modulus, with excellent comprehensive mechanical properties comparable to those of the benchmark alloy. Further exploration of the relationship between the primary alloying elements and the specific modulus of Al–Li alloys is required in future studies.

## Figures and Tables

**Table 1:** The range and contents of alloying elements in the dataset.

Elements	Elemental Contents Range/wt.%	Counts
Al	90.36~96.79	151
Li	0.49~3.78	151
Cu	0.04~4.7	134
Mg	0.015~5.9	133
Zn	0.001~1.31	67
Ti	0.01~0.6	39
Mn	0.005~0.86	91
Cr	0.0004~0.1	25
Zr	0.01~1	128
Fe	0.012~0.58	85
Si	0.0025~0.85	79
Sc	0.0433~0.31	13
Ag	0.005~0.62	61

**Table 2:** Twenty-six elemental characteristics’ parameters.

Definition	Descriptor	Unit	Symbol	Type
Number of Neutrons	N	-	NN	int
Atomic Number	Z	-	NA	int
Relative Atomic Mass	$A_{r}$	-	WA	float
Density	ρ	${g\cdot \mathrm{cm}}^{-2}$	D	float
Melting Temperature	$T_{m}$	°C	TM	float
Boiling Temperature	$T_{b}$	°C	TB	float
Valence Electrons	-	-	VE	float
Group Number in Periodic Table	G_n_	-	G	int
Period Number in Periodic Table	P_n_	-	P	int
Heat of Fusion	$∆H_{\mathrm{fus}}$	${\mathrm{kJ}\cdot \mathrm{mol}}^{-1}$	HF	float
Specific Heat Capacity	C	J/(kg·K)	SH	float
Coefficient of Thermal Expansion	α	°C^−1^	CTE	float
Enthalpy of Vaporization	$∆H_{\mathrm{vap}}$	${\mathrm{kJ}\cdot \mathrm{mol}}^{-1}$	VH	float
Ionic Charge	-	-	IC	float
Numbers of Valence Electrons	-	-	VEC	int
Atomic Ionization Energy	I	eV	EAI	float
Atomic Radius	$r_{a}$	pm	RA	float
Covalent Radius	$r_{c}$	pm	RC	float
Conductivity	κ	${S\cdot m}^{-1}$	C	float
Resistivity	R	Ω·m	R	float
Volume of Lattice	$V_{L}$	${\text{Å}}^{3}$	VL	float
Number of Space Group	-	-	NSG	int
Electronegative	χ	-	EN	float
Electron Affinity	EA	${\mathrm{kJ}\cdot \mathrm{mol}}^{-1}$	EA	float
The 1st Ionization Weight	$E_{i,\;1\mathrm{st}}$	${\mathrm{kJ}\cdot \mathrm{mol}}^{-1}$	1WI	float
The 2nd Ionization Weight	$E_{i,\;2\mathrm{nd}}$	${\mathrm{kJ}\cdot \mathrm{mol}}^{-1}$	2WI	float

**Table 3:** Solid solubility of elements M1 and M2 in aluminum.

M1/wt.%		M2/wt.%	
Zn	83.1	Au	0.44
Ag	53.56	Gd	0.4
Ga	19.95	Yb	0.3
Ge	5.54	Sb	0.28
Ho	5.2	Ni	0.24
Si	1.56	Nb	0.23
Sm	1.37	In	0.17
Mn	1.24	Nd	0.17
Ti	1.18	Sc	0.16
Ce	1.0	Bi	0.15
La	1.0	Zr	0.15
Cr	0.69	Be	0.1
Sn	0.64	Eu	0.1
Er	0.6	Pr	0.1
Sr	0.6	Y	0.1
V	0.58	W	0.08
Ca	0.5	Fe	0.05

**Table 4:** Prediction results of nominal composition and specific modulus of alloys.

Alloys	Nominal Elemental Content/wt.%								Specific Modulus GPa/(g·cm^−3^)
	Li	Cu	Mg	Zr	Ag	Mn	M		
1#	2.4	0.1	0.1	0.1	0.3	0.3	Ag	3.9	33.905
2#	2.3	0.1	0.4	0.1	0.3	0.3	Ho	3.7	33.986
3#	2.3	0.1	0.2	0.1	0.3	0.3	Ge	3.9	33.986
4#	2.4	0.3	1.2	0.1	0.3	0.3	Ge	2.6	33.264
5#	2.5	0.3	1.1	0.1	0.3	0.3	Zn	2.6	33.264
6#	2.4	1.3	1.7	0.1	0.3	0.3	Ga	1.1	33.137

**Table 5:** Casting sample specific modulus test results.

Alloys	M1	Density/(g·cm^−3^)	Specific Modulus /(GPa/(g·cm^−3^))	Error/%
1#	Ag	2.639	30.315	−10.59
2#	Ho	2.622	30.890	−9.11
3#	Ge	2.644	29.461	−13.31
4#	Ge	2.604	29.760	−10.53
5#	Zn	2.582	31.178	−6.27
6#	Ga	2.593	30.891	−6.78

**Table 6:** Homogenization regimes of casting sample.

Alloys	Homogenization Regimes
1#	540 °C/48 h
2#	540 °C/24 h
5#	540 °C/24 h
6#	530 °C/48 h

**Table 7:** Specific modulus and specific strength of composition-optimized alloys in aged condition.

Alloys	M1	Density/(g·cm^−3^)	Specific Modulus/(GPa/(g·cm^−3^))	Error/%
1#	Ag	2.633	30.803	−9.15
2#	Ho	2.634	31.620	−6.96
5#	Zn	2.582	31.526	−5.23
6#	Ga	2.591	31.531	−4.85
Benchmark alloy		2.625	31.691	/

## Data Availability

The raw/processed data required to reproduce these findings cannot be shared at this time due to technical or time limitations.
